# Circular RNA hsa_circ_0000690 as a potential biomarker for diagnosis and prognosis of intracranial aneurysm: Closely relating to the volume of hemorrhage

**DOI:** 10.1002/brb3.2929

**Published:** 2023-03-06

**Authors:** Yanming Huang, Huimin Cao, Xiaolong Qi, Celin Guan, Shuanglin Que

**Affiliations:** ^1^ Department of Neurosurgery Longyan First Affiliated Hospital of Fujian Medical University Longyan Fujian China; ^2^ Clinical Laboratory Longyan First Affiliated Hospital of Fujian Medical University Longyan Fujian China

**Keywords:** 3D slicer, biomarker, circRNA, diagnosis, intracranial aneurysm, prognosis, subarachnoid hemorrhage

## Abstract

**Purpose:**

This study aimed to explore circular RNA (circRNA) hsa_circ_0000690 as a potential biomarker for diagnosis and prognosis of intracranial aneurysm (IA) and its relationship with clinical factors and complications of IA.

**Material/methods:**

216 IA patients admitted to the neurosurgery department of our hospital from January 2019 to December 2020 were selected as the experimental group, and 186 healthy volunteers were selected as the control group. The expression of hsa_circ_0000690 in peripheral blood was detected by quantitative real‐time PCR, and its diagnostic value was assessed by receiver operating characteristic curve. Relationship between hsa_circ_0000690 and clinical factors of IA was assessed by chi‐square test. Nonparametric test was used in univariate analysis, and regression analysis was used in multivariate analysis. Multivariate Cox proportional hazards regression analysis was used to analyze the survival time.

**Results:**

CircRNA hsa_circ_0000690 of IA patients was relatively lower than that in the control group (*p* < .001). The AUC of hsa_circ_0000690 was 0.752, the specificity was 0.780, and sensitivity was 0.620, with diagnostic threshold of 0.0449. In addition, hsa_circ_0000690 expression was correlated with Glasgow Coma Scale, the volume of subarachnoid hemorrhage, modified Fisher scale, Hunt–Hess levels and surgical type. For hydrocephalus and delayed cerebral ischemia, hsa_circ_0000690 was significant in univariate analysis, but nonsignificant in multivariate analysis. For prognosis, hsa_circ_0000690 was significantly associated with modified Rankin Scales after surgery for 3 months, but not associated with survival time.

**Conclusions:**

The expression of hsa_circ_0000690 can act as a diagnostic marker for IA and predict the prognosis of 3 months after operation and is closely related to the volume of hemorrhage.

## Graphical abstract

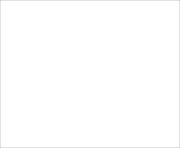

## 1 INTRODUCTION

Intracranial aneurysm (IA) is a cerebrovascular disease caused by high blood flow or congenital weakness of the cerebral artery wall (Marbacher et al., 2020), which seriously threatens human life and health. Currently, the diagnosis of IA mainly depends on digital subtraction angiography (DSA) and computed tomography angiography (CTA). Although brain imaging techniques and surgery strategies have been improved in past decades, the prognosis of subarachnoid hemorrhage (SAH) has not sufficiently improved (Algra et al., 2019). IA in most patients is asymptomatic until the aneurysm ruptures and pain appears and leads to SAH, which could be diagnosed and treated before rupture. Thus, early diagnosis and surgical treatment before rupture could significantly improve the prognosis of IAs.

Circular RNA (circRNA) is a type of novel nucleic acid molecule that exists in the form of a covalent loop, without a characteristic 5′cap end and 3′poly (A) end (Rybak‐Wolf et al., 2015). Present studies have shown that circRNAs have many functions, such as miRNA sponges, gene regulation, and close relation with cell functions and diseases. A large number of subsequent studies have confirmed that circRNAs involve many important pathological processes such as inflammation, SMC phenotype transformation, and extracellular matrix, which play important roles in epigenetic regulation in vascular diseases (Ashraf et al., 2021; Li et al., 2022). So, circRNAs have rapidly become popular in cerebrovascular disease research. Although DSA and CTA are dominating clinical tools for diagnosing IA, these methods are invasive and radioactive, which makes them unsuitable as a method of population screening. Peripheral blood is easy to collect, detecting that circulating circRNAs could be an efficient and effective method for the screening. Meanwhile the noninvasive and disease‐specific pattern is an essential feature of a biomarker. Since that, circRNAs have been deeply explored as a rising biological marker and therapeutic target of IA (Wu et al., 2021) and other diseases.

Based on our previous high‐throughput sequencing results (Cao et al., 2021), we first validated that hsa_circ_0000690 downregulated significantly in IA patients. Hsa_circ_0000690 locates at chr16:30495147‐30495584 in circBase with its associated‐gene symbol as integrin subunit αL (ITGAL). In this study, we explored the role of hsa_circ_0000690 in the diagnosis and prognosis of IA and its relationship with clinical factors and complications.

## 2 MATERIALS AND METHODS

### 2.1 Study population

A cohort of 216 cases of IA patients who were admitted in the department of neurosurgery in our hospital from January 2019 to December 2020 were collected as the experimental group, and 186 healthy volunteers over the same period served as the control group.

After admission, all IA patients underwent computed tomography (CT) examinations and diagnosed by CTA before surgery. Exclusion criteria were unruptured IAs, severe concomitant illness, or cardiovascular complications, incomplete clinical data.

The present study was approved by the Ethics Committee of Longyan First Affiliated Hospital of Fujian Medical University, and written informed consent was obtained from all participants.

Peripheral venous blood was drawn from IA patients or healthy volunteers with k2 EDTA‐coated vacutainer tubes. IA patients complete blood collection within 1 h after admission. About 2 mL of blood samples was centrifuged at 1500 *g*/min for 15 min to remove blood cells. The supernatant was got and transferred into an RNase‐free centrifuge tube and stored it at −80°C immediately for preparation of isolation of RNA subsequently.

### 2.2 Total RNA extraction

Total RNA was isolated from peripheral blood using QIAGEN serum/Plasma Kit, according to the kit's instructions. The NanoDrop ND‐2000 nucleic acid quantifier was used to determine the RNA content. The specimens with OD260/280 values between 1.80 and 2.00 were considered qualified samples.

### 2.3 cDNA synthesis and real‐time PCR

The cDNA was synthesized using the High‐Capacity cDNA Reverse Transcription Kit with RNase Inhibitor (ABI, USA) in accordance with the manufacturer's instructions. Real‐time PCR was performed using Power SYBR Green PCR Master Mix (ABI, USA) according to standard methods. The reaction conditions are as follows: pre‐denaturation 95°C (10 min), 1 cycle; PCR reaction 95°C (15 s), 60°C (1 min), 40 cycles; melting reaction 95°C (15 s), 60°C (1 min), 95°C (15 s), 1 cycle. GAPDH served as internal reference. The expression was calculated using the 2−ΔΔ*Ct* method. The primers used in this study are listed as follows: hsa_circ_0000690 F:5′‐TCATTGAGGGACAGAGGTGTT‐3′; R:5′‐GATAAGCACTTTGGTGGCATCTG‐3′; GAPDH F:5′‐TGACTTCAACAGCGACACCCA‐3′; R:5′‐CACCCTGTTGCTGTAGCCAAA‐3′.

### 2.4 Admission CT risk factors

A total of 216 brain CT data on admission were acquired in Digital Imaging and Communications in Medicine format (DICOM). All DICOM data were performed using a GE Discovery 750 CT scanner (General Electric Company, Fairfield, CT, USA,) with a slice thickness of 1.25 mm and an increment of 0.5 mm.

Based on these DICOM data, the modified Fisher scale (mFS) was computed for each patient according to Frontera et al. (2006). For a quantitative measure of aSAH and IVH volume, the DICOM data were transferred to a standard personal computer (Intel Core i5‐ 8250U CPU, 1.6 × 1.8 GHz, 8GB RAM) and then assessed by 3D Slicer (version 4.11.20200930) independently by two physicians. The Threshold Effect tool in Segment Editor module was applied to automatically mark the aSAH and the IVH. Manual drawing was then performed with the Paint Effect tool to prune or modify the colored structures slice by slice. Then a 3D model was constructed, and the volume was given by accumulating volume of all the pixels. We defined the result of aSAH and IVH volume as ‘Slicer Volume’ henceforth. For subsequent statistical analyses, the average of the two physicians’ Slicer Volume was obtained.

### 2.5 Non‐CT risk factors

Other non‐CT risk factors included demographic factors (gender, age), past history (smoking, drinking, hypertension, cardiac disease, and diabetes), admission clinical features (Hunt and Hess level (Ghosh et al., 2012), Glasgow Coma Scale (Avezaat et al., 1977)), admission radiological features (multiple aneurysms or not, position, and size), and surgical type. The position of the aneurysm was classified as anterior circulation or posterior circulation aneurysm. Size of the aneurysm was defined as small when diameter <5 mm, normal with diameter ≥5 and <15 mm, large with diameter ≥15 and <25 mm, giant with diameter ≥25 mm. Surgical type was divided into aneurysm clipping or embolism.

### 2.6 Definition of hydrocephalus and delayed cerebral ischemia (DCI)

Hydrocephalus was defined as ventricular enlargement, Evans’ index ≥.3 (Lee et al., 2021), and ventriculoperitoneal shunt was performed.

When potential causes of clinical deterioration, such as rebleeding, or seizures, were rigorously excluded, DCI was defined as (1) unexplained clinical deterioration (i.e., a new focal deficit, decrease in level of consciousness, or both) or (2) a new infarct on CT that was not visible on the admission or immediate postoperative scan, or both. DCI was diagnosed by the treating neurologist and confirmed in a retrospective review of each subject's clinical course by two physicians. Evidence of arterial spasm by transcranial doppler sonography was generally used to support the diagnosis but was not mandatory.

### 2.7 Prognosis and follow‐up

The prognosis after surgery for 3 months was determined by modified Rankin Scales (mRS). The follow‐up was from the hospital discharge time after the patient was treated to April 2022. For survival patients at the end of the follow‐up visit, the follow‐up data were the last contact state. For patients lost follow‐up, the follow‐up data were the last census state. The survival time was expressed by survival months.

### 2.8 Statistical analysis

All statistical analyses were performed with SPSS statistics 25 (IBM Corporation, USA). Mann–Whitney *U* was used to test whether IA patients and normal controls had the same distribution, including the difference in expression of hsa_circ_0000690. Receiver operating characteristic (ROC) curve was drawn by GraphPad Prism 8 to access the diagnosis value. The relationship between hsa_circ_0000690 and clinical factors was evaluated by chi‐square test. Mann–Whitney *U* or Kruskal–Wallis *H* were used for univariate analysis, whereas binary logistic regression or ordinal logistic regressions were used for multivariate analysis. The survival curve was drawn by Kaplan–Meier method and analyzed by long‐rank test. Multivariate cox proportional hazards regression analysis was used to analyze the risk factors in prognosis. *p* < .05 was considered statistically significant.

## 3 RESULTS

### 3.1 Study population

A total of 216 cases of IA patients included 73 males and 143 females. The age range was 28–83 years, and the average age was 58 years. The diameter of the aneurysm <5 mm in 82 cases, between 5 and 15 mm in 109 cases, between 15 and 25 mm in 24 cases, more than 25 mm in 1 case; anterior circulation aneurysms in 207 cases, posterior circulation aneurysms in 9 cases; single aneurysm in 188 cases, multiple aneurysms in 28 cases. IA patients and normal controls had the same distribution among gender, age, smoking, drinking, cardiac disease, and diabetes, expect hypertension. Hypertension was significantly higher in IA than that in normal controls (*p* < .001). See Table S1 for the results.

### 3.2 Down‐expression of hsa_circ_0000690 in IA patients

**FIGURE 1:**
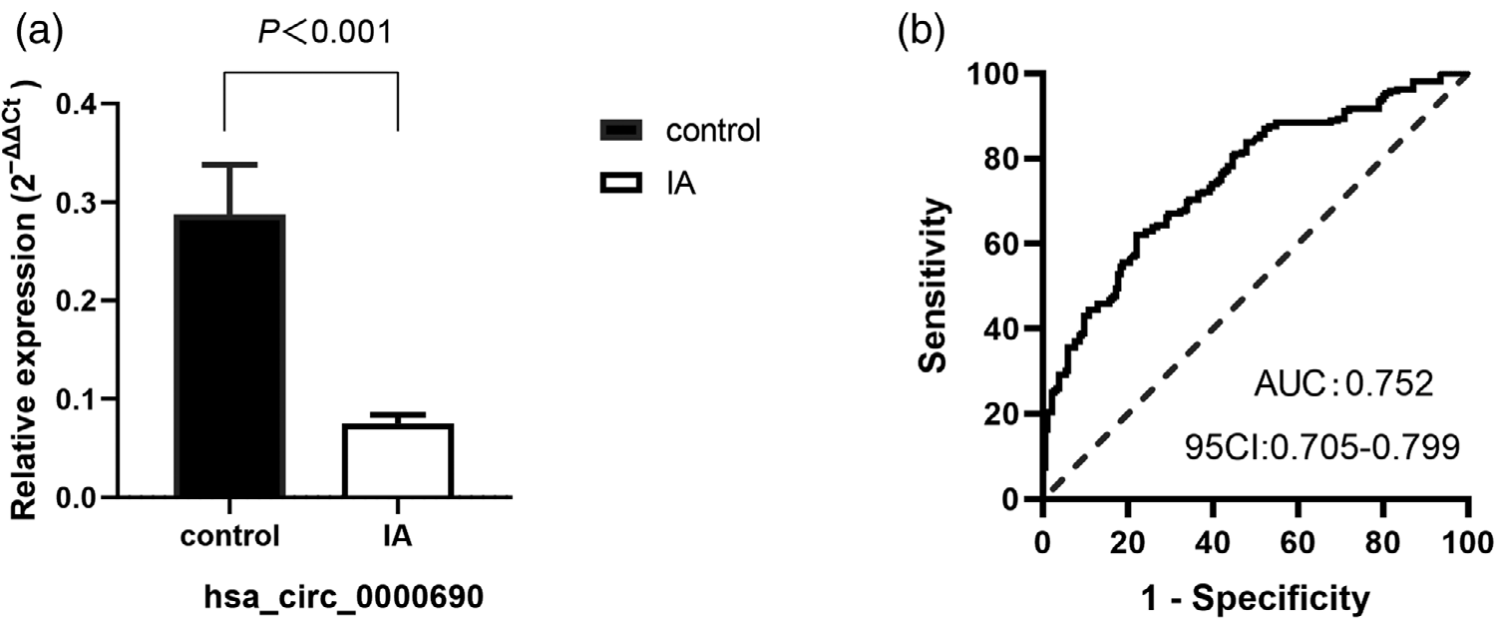
Quantitative real‐time PCR (qRT‐PCR) analysis and receiver operating characteristic (ROC) curve for hsa_circ_0000690 in intracranial aneurysm (IA) patients and control group. (A) The relative expression of hsa_circ_0000690 in IA patients is significantly lower than that in control group. (B)The ROC curve of hsa_circ_0000690.

Total RNA of peripheral blood was extracted from 216 IA patients and 186 normal controls and analyzed by quantitative real‐time PCR. Our results showed that expression of hsa_circ_0000690 in IA patients was significantly lower than that in normal controls (*p* < .001) (Figure 1A).

### 3.3 The potential diagnostic value of hsa_circ_0000690 for IA patients

The AUC of hsa_circ_0000690 ROC was 0.752 (95% confidence interval (CI): .705–.799). The specificity was 0.780, and sensitivity was 0.620, with diagnostic threshold of 0.0449 (Figure 1B).

### 3.4 Relationship between expression of hsa_circ_0000690 and clinical features in IA patients

As shown in Table 1, hsa_circ_0000690 expression was significantly associated with Glasgow Coma Scale (GCS), Slicer Volume, mFS, Hunt–Hess levels, and surgical type (*p* < .05).

**TABLE 1:** Relationship between expression of hsa_circ_0000690 and clinical factors

Clinical factors	*n* = 216	Low hsa_circ_0000690 expression [*n* (%)]	High hsa_circ_0000690 expression [*n* (%)]	*χ* ^2^	*p*‐Value
**Gender**				.146	.703
**Female**	143	90 (62.9)	53 (37.1)		
**Male**	73	44 (60.3)	29 (39.7)		
**Age**				.199	.656
**≤55 year**	99	63 (63.6)	36 (36.4)		
**>55 year**	117	71 (60.7)	46 (39.3)		
**Smoking**				2.696	.101
**No**	173	112 (64.7)	61 (35.3)		
**Yes**	43	22 (51.2)	21 (48.8)		
**Drinking**				2.658	.103
**No**	180	116 (64.4)	64 (35.6)		
**Yes**	36	18 (50.0)	18 (50.0)		
**Hypertension**				.009	.923
**No**	115	71 (61.7)	44 (38.3)		
**Yes**	101	63 (62.4)	38 (37.6)		
**Cardiac disease**				1.862	.172
**No**	213	131 (61.5)	82 (38.5)		
**Yes**	3	3 (100)	0 (0)		
**Diabetes**				1.507	.220
**No**	194	123 (63.4)	71 (36.6)		
**Yes**	22	11 (50.0)	11 (50.0)		
**Slicer Volume**				73.158	<.001^**^
**≤18** **mL**	112	39 (34.8)	73 (65.2)		
**>18** **mL**	104	95 (91.3)	9 (8.7)		
**mFS**				69.512	<.001^**^
**I**	47	10 (21.3)	37 (78.7)		
**II**	52	23 (44.2)	29 (55.8)		
**III**	47	40 (85.1)	7 (14.9)		
**IV**	70	61 (87.1)	9 (12.9)		
**Hunt–Hess**				37.216	<.001^**^
**I**	15	3 (20.0)	12 (80.0)		
**II**	78	38 (48.7)	40 (51.3)		
**III**	81	53 (65.4)	28 (34.6)		
**IV**	39	37 (94.9)	2 (5.1)		
**V**	3	3 (100)	0 (0)		
**GCS**				25.510	<.001^**^
**3–8**	44	41 (93.2)	3 (6.8)		
**9–12**	33	22 (66.7)	11 (33.3)		
**13–15**	139	71 (51.1)	68 (48.9)		
**Multiple aneurysms or not**				.979	.322
**Single**	188	119 (63.3)	69 (36.7)		
**Multiple**	28	15 (53.6)	13 (46.4)		
**Aneurysm position**				1.234	.267
**Anterior circulation aneurysm**	207	130 (62.8)	77 (37.2)		
**Posterior circulation aneurysm**	9	4 (44.4)	5 (55.6)		
**Aneurysm size**				2.477	.479
**0–0.5** **cm**	82	50 (61.0)	32 (39.0)		
**0.5–1.5** **cm**	109	67 (61.5)	42 (38.5)		
**1.5–2.5** **cm**	24	17 (70.8)	7 (29.2)		
**≥2.5** **cm**	1	0 (0)	1 (100)		
**Surgery**				9.937	.002^**^
**Embolism**	77	37 (48.1)	40 (51.9)		
**Clipping**	139	97 (69.8	42 (30.2)		

Abbreviations: GCS, Glasgow Coma Scale; mFS, modified Fisher scale.

^*^ *p* < .05.

^**^ *p* < .01.

### 3.5 Relationship between expression of hsa_circ_0000690 and hydrocephalus in IA patients

IA patients were grouped according to whether they had hydrocephalus, and the univariate analysis showed that the expressions of hsa_circ_0000690, gender, age, smoking, Slicer Volume, mFS, Hunt–Hess levels, GCS, aneurysm size, and surgical type were significantly different between the two groups (*p* < .05) (Table S2). But through binary logistic regression for significant factors mentioned above (Table 2), hydrocephalus was only significantly associated with smoking, Slicer Volume, and aneurysm size, without an association with the expression of hsa_circ_0000690.

**TABLE 2:** Relationship between clinical factors and hydrocephalus in intracranial aneurysm (IA) patients by binary logistic regression

Clinical factors						95% CI	
	*B*	SE	Wald	OR	*p*‐Value	Lower	Upper
**hsa_circ_0000690**	−3.799	5.829	0.425	0.022	.515	.000	2051.190
**Gender**	−0.796	0.604	1.736	0.451	.188	.138	1.474
**Age**	0.025	0.019	1.715	1.025	.190	.988	1.064
**Smoking**	−2.367	1.001	5.587	0.094	.018^*^	.013	.667
**Slicer Volume**	0.054	0.019	7.855	1.055	.005^**^	1.016	1.096
**mFS**	0.176	0.286	0.377	1.192	.539	.680	2.089
**Hunt–Hess**	0.451	0.564	0.640	1.570	.424	.520	4.741
**GCS**	−0.079	0.115	0.474	0.924	.491	.737	1.158
**Aneurysm size**	1.031	0.359	8.250	2.805	.004^**^	1.388	5.671
**Surgery**	0.305	0.508	0.360	1.356	.548	.501	3.667

Abbreviations: CI, confidence interval; GCS, Glasgow Coma Scale; mFS, modified Fisher scale.

^*^ *p* < .05.

^**^ *p* < .01.

### 3.6 Relationship between expressions of hsa_circ_0000690 and DCI in IA patients

IA patients were grouped according to whether they had DCI, and the univariate analysis showed that the expressions of hsa_circ_0000690, age, diabetes, Slicer Volume, mFS, Hunt–Hess levels, GCS, and surgical type were significantly different between the two groups (*p* < .05) (Table S3). But through binary logistic regression for significant factors mentioned above (Table 3), DCI was only significantly associated with age, diabetes, mFS, and surgical type, without an association with the expression of hsa_circ_0000690.

**TABLE 3:** Relationship between clinical factors and delayed cerebral ischemia in intracranial aneurysm (IA) patients by binary logistic regression

Clinical factors						95% CI	
	*B*	SE	Wald	OR	*p*‐Value	Lower	Upper
**hsa_circ_0000690**	1.326	1.547	0.735	3.767	.391	.182	78.171
**Age**	0.034	0.014	5.925	1.035	.015^*^	1.007	1.063
**Diabetes**	1.234	0.574	4.621	3.434	.032^*^	1.115	10.573
**Slicer Volume**	0.020	0.015	1.850	1.020	.174	.991	1.051
**mFS**	0.604	0.208	8.438	1.829	.004^**^	1.217	2.749
**Hunt–Hess**	0.477	0.366	1.698	1.611	.192	.786	3.301
**GCS**	0.088	0.090	0.961	1.092	.327	.916	1.302
**Surgery**	0.907	0.367	6.122	2.478	.013^*^	1.208	5.085

Abbreviations: CI, confidence interval; GCS, Glasgow Coma Scale; mFS, modified Fisher scale.

^*^ *p* < .05.

^**^ *p* < .01.

### 3.7 Relationship between expressions of hsa_circ_0000690 and mRS in IA patients

mRS was significantly associated with expression of hsa_circ_0000690, age, Slicer Volume, mFS, Hunt–Hess levels, GCS, aneurysm size, surgical type, hydrocephalus, and DCI (*p* < .05) through Kruskal–Wallis *H* test (Table S4). Through ordinal logistic regression for significant factors mentioned above (Table 4), mRS was significantly associated with expression of hsa_circ_0000690, Slicer Volume, mFS, and Hunt–Hess levels.

**TABLE 4:** Relationship between clinical factors and modified Rankin Scales in intracranial aneurysm (IA) patients by ordinal logistic regression

Clinical factors					95% CI	
	*B*	SE	Wald	*p*‐Value	Lower	Upper
**hsa_circ_0000690**	−5.500	1.929	8.128	.004^**^	−9.281	−1.719
**Age**	0.003	0.007	0.224	.636	−.011	.017
**Slicer Volume**	0.064	0.009	52.031	<.001^**^	.047	.081
**mFS**	0.231	0.110	4.368	.037^*^	.014	.447
**Hunt–Hess**	1.080	0.216	24.922	<.001^**^	.656	1.504
**GCS**	0.050	0.048	1.063	.302	−.045	.144
**Aneurysm size**	−0.008	0.133	0.003	.955	−.268	.253
**Surgery**	−0.227	0.193	1.379	.240	−.606	.152
**Hydrocephalus**	0.153	0.233	0.431	.511	−.304	.609
**DCI**	−0.148	0.195	0.578	.447	−.530	.234

Abbreviations: CI, confidence interval; GCS, Glasgow Coma Scale; mFS, modified Fisher scale.

^*^ *p* < .05.

^**^ *p* < .01.

### 3.8 Results of survival and risk factors

According to Kaplan–Meier curve (Table S5), survival times were significantly shorter in IA patients with lower expression of hsa_circ_0000690, higher Slicer Volume, higher mFS, higher Hunt–Hess levels, lower GCS, larger aneurysm size, clipping, hydrocephalus, and DCI. The Cox regression model with significant factors mentioned above showed that only hydrocephalus and DCI were the prognostic risk factors for IA (Table 5).

**TABLE 5:** Risk factors of intracranial aneurysm (IA) patients for prognosis by multivariate Cox proportional hazards regression analysis

						95% CI	
	*B*	SE	Wald	OR	*p*‐Value	Lower	Upper
**hsa_circ_0000690**	−0.824	1.099	0.562	0.439	.453	.051	3.781
**Slicer Volume**	0.866	0.540	2.567	2.377	.109	.824	6.854
**mFS**	0.146	0.248	0.346	1.157	.557	.712	1.880
**Hunt–Hess**	−0.225	0.426	0.278	0.799	.598	.347	1.840
**GCS**	−0.546	0.402	1.843	0.580	.175	.264	1.274
**Aneurysm size**	0.093	0.249	0.139	1.097	.709	.674	1.788
**Surgery**	−0.030	0.386	0.006	0.970	.938	.456	2.067
**Hydrocephalus**	0.827	0.416	3.947	2.287	.047^*^	1.011	5.173
**DCI**	0.905	0.415	4.762	2.472	.029^*^	1.097	5.571

Abbreviations: CI, confidence interval; GCS, Glasgow Coma Scale; mFS, modified Fisher scale.

^*^ *p* < .05.

^**^ *p* < .01.

## 4 DISCUSSION

circRNAs have good structural stability and been abundant in brain tissue, making the exploration of circRNAs as biological markers widely concerned and deeply studied. circRNAs play roles in different kinds of neurological disease, such as glioma (Sun et al., 2020), brain development diseases (Mehta et al., 2020), neurological traumatic injury (Xu et al., 2021; Zhang et al., 2022), and IA (Wu et al., 2021).

In our previous study (Cao et al., 2021) about circRNAs expression profile in multiple IAs (MIAs), we found that hsa_circ_0000690 was lower expression in MIAs than healthy people and played an important role in inflammatory response and leukocyte infiltration. Therefore, we enlarged the samples in this study to validate the expression difference of hsa_circ_0000690 and tried to find its potential diagnostic and prognostic value for IA, including single and MIAs. As a result, the expression of hsa_circ_0000690 in 216 IA patients was significantly lower than that in 186 normal controls (*p* < .001). Moreover, it showed potential diagnostic value for IA through ROC curve that the significant AUC of hsa_circ_0000690 was 0.752, and diagnostic threshold was 0.0449, with 0.780 in specificity and 0.620 in sensitivity, respectively.

In a systematic review and meta‐analysis (Wu et al., 2021) about noncoding RNAs as circulating biomarkers for the diagnosis of IA in 2021, most of noncoding RNAs for the diagnosis of IA were micro RNAs. There were only three circRNAs (Huang et al., 2019; Teng et al., 2017) having diagnostic value, which called hsa_circ_0021001, hsa_circ_0072309, and hsa_circ_0008433. Interestingly, the same as three circRNAs mentioned above, hsa_circ_0000690 is also downregulated and involved in inflammation and cell adhesion pathway, which are closely related to vascular strength and integrity. It is known that inflammation is an important factor in the occurrence and development of IA. Meanwhile most dysfunctional circRNAs are mainly involved in inflammation and cell adhesion pathways that are known to be critical for the pathogenesis of IA (Pawlowska et al., 2018). Inflammatory response (Signorelli et al., 2018; Turjman et al., 2014) and leukocyte infiltration (Kanematsu et al., 2011) are the pathological basis for the development of IA. Infiltration of inflammatory cells is the primary pathological change during the early stages of IA (Pawlowska et al., 2018). Reported in many research studies (Chu et al., 2015; Kim et al., 2014; Rothoerl et al., 2006), intercellular cell adhesion molecule‐1 (ICAM‐1) plays an important role in aneurysm formation and rupture. Then, adhesion between ICAM‐1 and lymphocyte function‐associated antigen‐1 (LFA‐1) is involved in ‘leukocyte transendothelial migration pathway’ (Figure S1A) and ‘natural killer cell mediated cytotoxicity pathway’ (Figure S1B), which are two important pathways in inflammation. One subunit of LFA‐1 is encoded by ITGAL, which is transcriptionally regulated by hsa_circ_0000690. This may be explained why hsa_circ_0000690 could be diagnostic biomarker for IA.

We also explored relationship between hsa_circ_0000690 and clinical features in IA. It was surprised to find IA patients with lower expression of hsa_circ_0000690 would have larger hematoma volume, higher level of mFS and Hunt–Hess but lower GCS scores. Furthermore, the most of IA patients (98.1%) with larger Slicer Volume would express lower level of hsa_circ_0000690. Slicer Volume is the volume of aSAH and IVH measured by 3D Slicer pixel by pixel. The accuracy of this software to measure the volume of an irregular hematoma has been demonstrated (Xu et al., 2014). It meant that hsa_circ_0000690 would be closely related to the volume of hemorrhage on admission. As larger hematoma volume was in lower expression of hsa_circ_0000690 group, it may explain that higher level of mFS, Hunt–Hess, and lower GCS scores were also in lower expression of hsa_circ_0000690 group. It assumed that hsa_circ_0000690 took part in the formation, development and rupture of IA through inflammatory response and leukocyte infiltration. And then, the lower the expression of hsa_circ_0000690, the more intensive inflammatory response and leukocyte infiltration, leading to easier rupture of IA and larger volume of hematoma.

The volume of hematoma on admission was also closely associated with complication and prognosis. Thick aSAH often caused early hydrocephalus and heavy DCI, leading to poor prognosis. Closely related to the volume of hemorrhage on admission, could hsa_circ_0000690 have value in complications and prognosis? What a pity! hsa_circ_0000690 was related to hydrocephalus and DCI in univariate analysis, but having no relationship with both of them in multivariate analysis. Although hsa_circ_0000690 had weak predictive value of complications, it had strong relationship with mRS both in univariate analysis and multivariate analysis. The IA patients with lower expression of hsa_circ_0000690 would have higher mRS score, indicating poor prognosis and living quality. For survival time of IA patients, hsa_circ_0000690 was significant in long‐rank test, but nonsignificant in multivariate cox proportional hazards regression analysis. It meant that the expression of hsa_circ_0000690 could predict prognosis and living quality after surgery for 3 months but could not be able to predict complications and survival time.

Possible limitations in this study also deserve mention. First, we found hsa_circ_0000690 would be closely related to the volume of hemorrhage on admission and offered an assumption, but due to the limitations of case–control studies, it is not yet possible to accurately determine the causal relationship between the circRNA and volume of hemorrhage. So, further functional tests will be needed to verify. Second, an advantage of this study is that volume of hemorrhage was measured by 3D Slicer, but it would be better to calculate hemorrhage in subarachnoid and ventricle separately. Third, because of none unruptured aneurysm in experimental group, it is no clear about the difference of hsa_circ_0000690 between unruptured IA patients and normal people. Moreover, function of hsa_circ_0000690 in progress of unruptured aneurysm, which would be further explored in our future work.

## 5 CONCLUSION

In conclusion, we introduced hsa_circ_0000690 as a potential novel biomarker for IA can predict the prognosis of 3 months after operation. Moreover, this is the first circRNA found to have closely relationship with the volume of hemorrhage when IA is ruptured as we know. We will explore its regulatory mechanism in the formation, development, and rupture of IA through further functional tests in the future, in order to provide a new perspective for the future research of IA noninvasive diagnosis and treatment strategies.

## AUTHOR CONTRIBUTIONS

Yanming Huang and Shuanglin Que contributed to the study conception and design. PCR experiment was performed by Huimin Cao. Clinical material preparation and data collection were performed by Yanming Huang, Xiaolong Qi, and Celin Guan. Data analysis and the first draft of the manuscript were written by Yanming Huang and Huimin Cao, whereas all authors commented on previous versions of the manuscript. All authors read and approved the final manuscript.

## CONFLICT OF INTEREST STATEMENT

The authors declare that they have no known conflict of interests or personal relationships that could have appeared to influence the work reported in this paper.

## CONSENT FOR PUBLICATION

The manuscript is not submitted for publication or consideration elsewhere.

### PEER REVIEW

The peer review history for this article is available at https://publons.com/publon/10.1002/brb3.2929.

## Supporting information

Supporting Information

## Data Availability

All data generated or analyzed during this study are included in this published article.
