# Supplementary material for: Circular RNA hsa_circ_0000690 as a potential biomarker for diagnosis and prognosis of intracranial aneurysm: Closely relating to the volume of hemorrhage
Source: Brain Behav. 2023 Mar 6;13(4):e2929. doi: 10.1002/brb3.2929 (PMC10097068; doi:10.1002/brb3.2929)
Supplement: Supplementary file 1 — Supporting Information [file BRB3-13-e2929-s001.docx]

Supplementary Material

# Supplementary Tables

| Supplementary Table 1. Distribution between IA patients and controls | | | | |
| --- | --- | --- | --- | --- |
|  | control=186 | ICA=216 | Z | p-value |
| Gender (female) | 109(58.6%) | 143(66.2%) | -1.569 | 0.117 |
| Age (≤55 year) | 74(39.8%) | 99(45.8%) | 0.371 | 0.711 |
| Smoking | 24(12.9%) | 43(19.9%) | 1.877 | 0.061 |
| Drinking | 27(14.5%) | 36(16.7%) | 0.591 | 0.555 |
| Hypertension | 27(14.5) | 101(46.8%) | 6.911 | ＜0.001** |
| Cardiac disease | 3(1.6%) | 3(1.4%) | -0.184 | 0.854 |
| Diabetes | 18(9.7%) | 22(10.2%) | 0.169 | 0.866 |
| *P < 0.05; **P < 0.01; | |  |  |  |

| **Supplementary Table 2. Relationship between clinical factors and hydrocephalus in IA patients by Mann-Whitney U test** | | | | |
| --- | --- | --- | --- | --- |
| **clinical factors** | **no=161** | **hydrocephalus=55** | **Z** | **p-value** |
| **hsa_circ_0000690** | 0.092±0.011 | 0.025±0.004 | -5.772 | ＜0.001 ** |
| **Gender (female)** | 99(61.5%) | 44(80.0%) | -2.500 | 0.012 * |
| **Age (≤55 year)** | 78(48.4%) | 21(38.2%) | 2.228 | 0.026 * |
| **Smoking** | 41(25.5%) | 2(3.6%) | -3.492 | ＜0.001 ** |
| **Drinking** | 31(19.3%) | 5(9.1%) | -1.742 | 0.081 |
| **Hypertension** | 74(46.0%) | 27(49.1%) | 0.400 | 0.689 |
| **Cardiac disease** | 1(0.6%) | 2(3.6%) | 1.646 | 0.100 |
| **Diabetes** | 16(9.9%) | 6(10.9%) | 0.205 | 0.837 |
| **SlicerVolume (≤18ml)** | 102(63.4%) | 10(18.2%) | 7.043 | ＜0.001 ** |
| **mFS** |  |  | 4.768 | ＜0.001 ** |
| **I** | 44(27.3%) | 3(5.5%) |  |  |
| **II** | 46(28.6%) | 6(10.9%) |  |  |
| **III** | 29(18.0%) | 18(32.7%) |  |  |
| **IV** | 42(26.1%) | 28(50.9%) |  |  |
| **Hunt-Hess** |  |  | 7.105 | ＜0.001 ** |
| **I** | 15(9.3%) | 0(0.0%) |  |  |
| **II** | 73(45.4%) | 5(9.1%) |  |  |
| **III** | 58(36.0%) | 23(41.8%) |  |  |
| **IV** | 15(9.3%) | 24(43.6%) |  |  |
| **V** | 0(0.0%) | 3(5.5%) |  |  |
| **GCS** |  |  | -7.382 | ＜0.001 ** |
| **3~8** | 16(9.9%) | 28(50.9%) |  |  |
| **9~12** | 19(11.8%) | 14(25.5%) |  |  |
| **13~15** | 126(78.3%) | 13(23.6%) |  |  |
| **Multiple or not** |  |  | -0.060 | 0.952 |
| **single** | 140(87.0%) | 48(87.3%) |  |  |
| **multiple** | 21(13.0%) | 7(12.7%) |  |  |
| **Aneurysm position** |  |  | 0.552 | 0.581 |
| **Anterior circulation aneurysm** | 155(96.3%) | 52(94.5%) |  |  |
| **Posterior circulation aneurysm** | 6(3.7%) | 3(5.5%) |  |  |
| **Aneurysm size** |  |  | 4.087 | ＜0.001 ** |
| **0~0.5cm** | 72(44.8%) | 10(18.2%) |  |  |
| **0.5~1.5cm** | 77(47.8%) | 32(58.2%) |  |  |
| **1.5cm~2.5cm** | 11(6.8%) | 13(23.6%) |  |  |
| **≥2.5cm** | 1(0.6%) | 0(0.0%) |  |  |
| **Surgery** |  |  | 3.311 | 0.002 ** |
| **Embolism** | 67(41.6%) | 10(18.2%) |  |  |
| **Clipping** | 94(58.4%) | 45(81.8%) |  |  |
| *P < 0.05; **P < 0.01 |  |  |  |  |

| **Supplementary Table 3. Relationship between clinical factors and delayed cerebral ischemia in IA patients by Mann-Whitney U test** | | | | |
| --- | --- | --- | --- | --- |
| **clinical factors** | **no=124** | **DCI=92** | **Z** | **p-value** |
| **hsa_circ_0000690** | 0.093±0.013 | 0.050±0.011 | -4.620 | ＜0.001 ** |
| **Gender (female)** | 79(63.7%) | 64(69.6%) | -0.898 | 0.369 |
| **Age (≤55 year)** | 65(52.4%) | 34(37.0%) | 2.469 | 0.014 * |
| **Smoking** | 28(22.6%) | 15(16.3%) | -1.140 | 0.254 |
| **Drinking** | 22(17.7%) | 14(15.2%) | -0.491 | 0.623 |
| **Hypertension** | 54(43.5%) | 47(51.1%) | 1.095 | 0.273 |
| **Cardiac disease** | 1(0.8%) | 2(2.2%) | 0.847 | 0.397 |
| **Diabetes** | 7(5.6%) | 15(16.3%) | 2.555 | 0.011 * |
| **SlicerVolume(≤18ml)** | 80(64.5%) | 32(34.8%) | 5.225 | ＜0.001 ** |
| **mFS** |  |  | 5.456 | ＜0.001 ** |
| **I** | 36(29.0%) | 11(12.0%) |  |  |
| **II** | 41(33.1%) | 11(12.0%) |  |  |
| **III** | 23(18.5%) | 24(26.0%) |  |  |
| **IV** | 24(1.4%) | 46(50.0%) |  |  |
| **Hunt-Hess** |  |  | 4.495 | ＜0.001 ** |
| **I** | 10(8.1%) | 5(5.4%) |  |  |
| **II** | 55(44.3%) | 23(25.0%) |  |  |
| **III** | 50(40.3%) | 31(33.7%) |  |  |
| **IV** | 9(7.3%) | 30(32.6%) |  |  |
| **V** | 0(0.0%) | 3(3.3%) |  |  |
| **GCS** |  |  | -2.947 | 0.003 ** |
| **3~8** | 10(8.0%) | 34(37.0%) |  |  |
| **9~12** | 25(20.2%) | 8(8.7%) |  |  |
| **13~15** | 89(71.8%) | 50(54.3%) |  |  |
| **Multiple or not** |  |  | -1.196 | 0.232 |
| **single** | 105(84.7%) | 83(90.2%) |  |  |
| **multiple** | 19(15.3%) | 9(9.8%) |  |  |
| **Aneurysm position** |  |  | -0.573 | 0.567 |
| **Anterior circulation aneurysm** | 118(95.2%) | 89(96.7%) |  |  |
| **Posterior circulation aneurysm** | 6(4.8%) | 3(3.3%) |  |  |
| **Aneurysm size** |  |  | -0.156 | 0.876 |
| **0~0.5cm** | 49(39.5%) | 33(35.9%) |  |  |
| **0.5~1.5cm** | 57(46.0%) | 52(56.5%) |  |  |
| **1.5cm~2.5cm** | 17(13.7%) | 7(7.6%) |  |  |
| **≥2.5cm** | 1(0.8%) | 0(0.0%) |  |  |
| **Surgery** |  |  | 3.026 | 0.002 ** |
| **Embolism** | 55(44.4%) | 22(23.9%) |  |  |
| **Clipping** | 69(55.6%) | 70(76.1%) |  |  |
| *P < 0.05; **P < 0.01 |  |  |  |  |

| **Supplementary Table 4. Relationship between clinical factors and modified Rankin Scales in IA patients by Kruskal-Wallis H** | | | | | | | | | |
| --- | --- | --- | --- | --- | --- | --- | --- | --- | --- |
| **clinical factors** | **mean rank** | | | | | | |  |  |
|  | **mRS0=53** | **mRS1=35** | **mRS2=33** | **mRS3=39** | **mRS4=18** | **mRS5=22** | **mRS6=16** | **H** | **p-value** |
| **hsa_circ_0000690** | 164.62 | 159.59 | 107.03 | 76.73 | 40.97 | 48.27 | 50.09 | 131.641 | ＜0.001 ** |
| **Gender** | 110.72 | 112.11 | 101.45 | 105.23 | 120.00 | 111.27 | 99.00 | 2.581 | 0.859 |
| **Age** | 105.48 | 111.84 | 114.36 | 103.60 | 81.42 | 100.27 | 152.81 | 12.570 | 0.05 * |
| **Smoking** | 115.53 | 111.69 | 106.64 | 103.62 | 111.00 | 106.64 | 93.75 | 4.115 | 0.661 |
| **Drinking** | 114.95 | 109.01 | 103.59 | 98.81 | 120.50 | 115.05 | 97.25 | 7.517 | 0.276 |
| **Hypertension** | 98.75 | 107.37 | 113.64 | 113.38 | 106.00 | 107.09 | 125.50 | 3.997 | 0.677 |
| **Cardiac disease** | 109.04 | 107.00 | 107.00 | 109.77 | 107.00 | 107.00 | 113.75 | 4.749 | 0.576 |
| **Diabetes** | 109.73 | 106.76 | 104.05 | 103.04 | 109.50 | 117.14 | 117.75 | 4.694 | 0.584 |
| **SlicerVolume** | 40.81 | 66.14 | 108.30 | 138.62 | 178.61 | 179.14 | 176.38 | 156.928 | ＜0.001 ** |
| **mFS** | 54.57 | 78.77 | 110.45 | 131.54 | 156.00 | 163.30 | 163.22 | 99.090 | ＜0.001 ** |
| **Hunt-Hess** | 46.97 | 89.13 | 113.55 | 124.38 | 159.58 | 165.91 | 169.16 | 115.355 | ＜0.001 ** |
| **GCS** | 159.75 | 134.23 | 110.73 | 87.59 | 55.25 | 63.16 | 51.06 | 88.977 | ＜0.001 ** |
| **Multiple or not** | 104.69 | 119.19 | 110.86 | 105.58 | 100.50 | 104.32 | 114.75 | 5.632 | 0.466 |
| **Aneurysm position** | 112.15 | 104.00 | 107.27 | 106.77 | 110.00 | 108.91 | 110.75 | 3.648 | 0.724 |
| **Aneurysm size** | 98.30 | 92.51 | 121.03 | 125.41 | 128.94 | 99.64 | 99.38 | 12.990 | 0.043 * |
| **Surgery** | 98.09 | 97.63 | 97.91 | 113.77 | 129.00 | 122.45 | 133.50 | 13.581 | 0.035 * |
| **Hydrocephalus** | 85.08 | 90.26 | 100.64 | 117.00 | 135.00 | 139.91 | 148.50 | 47.451 | ＜0.001 ** |
| **DCI** | 84.92 | 105.70 | 91.95 | 109.58 | 116.50 | 145.95 | 163.75 | 41.773 | ＜0.001 ** |
| *P < 0.05; **P < 0.01 |  |  |  |  |  |  |  |  |  |

| **Supplementary Table 5. Survival time of IA patients according to clinical factors by Kaplan-Meier analysis** | | | | | | | |
| --- | --- | --- | --- | --- | --- | --- | --- |
|  |  |  |  | **95% CI** | |  |  |
|  | **n=216** | **estimate months** | **Std.Error** | **Lower** | **Upper** | **χ2** | **p-value** |
| **hsa_circ_0000690** |  |  |  |  |  | 12.015 | 0.001** |
| **low hsa_circ_0000690 expression** | 134 | 27.489 | 1.252 | 25.036 | 29.943 |  |  |
| **high hsa_circ_0000690 expression** | 82 | 33.870 | 0.954 | 31.999 | 35.741 |  |  |
| **Gender** |  |  |  |  |  | 0.001 | 0.973 |
| **Female** | 143 | 29.893 | 1.086 | 27.765 | 32.022 |  |  |
| **Male** | 73 | 29.950 | 1.520 | 26.970 | 32.930 |  |  |
| **Age** |  |  |  |  |  | 3.601 | 0.058 |
| **≤55 year** | 99 | 31.705 | 1.138 | 29.475 | 33.934 |  |  |
| **＞55 year** | 117 | 28.390 | 1.300 | 25.841 | 30.939 |  |  |
| **Smoking** |  |  |  |  |  | 1.967 | 0.161 |
| **No** | 173 | 29.266 | 1.025 | 27.258 | 31.274 |  |  |
| **Yes** | 43 | 32.518 | 1.583 | 29.415 | 35.621 |  |  |
| **Drinking** |  |  |  |  |  | 0.000 | 0.986 |
| **No** | 180 | 29.888 | 0.973 | 27.981 | 31.794 |  |  |
| **Yes** | 36 | 28.952 | 2.020 | 24.993 | 32.911 |  |  |
| **Hypertension** |  |  |  |  |  | 2.822 | 0.093 |
| **No** | 115 | 31.285 | 1.086 | 29.157 | 33.413 |  |  |
| **Yes** | 101 | 28.330 | 1.416 | 25.556 | 31.105 |  |  |
| **Cardiac disease** |  |  |  |  |  | 0.424 | 0.515 |
| **No** | 213 | 29.983 | 0.885 | 28.248 | 31.719 |  |  |
| **Yes** | 3 | 16.863 | 6.097 | 4.914 | 28.812 |  |  |
| **Diabetes** |  |  |  |  |  | 2.770 | 0.096 |
| **No** | 194 | 30.393 | 0.903 | 28.624 | 32.163 |  |  |
| **Yes** | 22 | 24.241 | 3.044 | 18.275 | 30.207 |  |  |
| **SlicerVolume** |  |  |  |  |  | 29.656 | ＜0.001 ** |
| **≤18ml** | 112 | 34.528 | 0.704 | 33.147 | 35.908 |  |  |
| **＞18ml** | 104 | 24.959 | 1.521 | 21.979 | 27.940 |  |  |
| **mFS** |  |  |  |  |  | 21.986 | ＜0.001 ** |
| **I** | 47 | 34.877 | 0.978 | 32.961 | 36.794 |  |  |
| **II** | 52 | 32.939 | 1.110 | 30.763 | 35.115 |  |  |
| **III** | 47 | 25.889 | 2.004 | 21.960 | 29.818 |  |  |
| **IV** | 70 | 25.640 | 1.789 | 22.133 | 29.147 |  |  |
| **Hunt-Hess** |  |  |  |  |  | 43.736 | ＜0.001 ** |
| **I** | 15 | 33.683 | 1.784 | 30.187 | 37.180 |  |  |
| **II** | 78 | 34.699 | 0.782 | 33.167 | 36.231 |  |  |
| **III** | 81 | 28.586 | 1.419 | 25.805 | 31.367 |  |  |
| **IV** | 39 | 16.999 | 2.187 | 12.712 | 21.285 |  |  |
| **V** | 3 | 10.663 | 4.219 | 2.395 | 18.932 |  |  |
| **GCS** |  |  |  |  |  | 46.716 | ＜0.001 ** |
| **3~8** | 44 | 18.215 | 2.305 | 13.698 | 22.732 |  |  |
| **9~12** | 33 | 24.400 | 2.863 | 18.788 | 30.011 |  |  |
| **13~15** | 139 | 33.641 | 0.738 | 32.196 | 35.087 |  |  |
| **Multiple or not** |  |  |  |  |  | 0.141 | 0.707 |
| **Single** | 188 | 29.775 | 0.958 | 27.896 | 31.653 |  |  |
| **Multiple** | 28 | 29.482 | 2.193 | 25.183 | 33.780 |  |  |
| **Aneurysm position** |  |  |  |  |  | 1.682 | 0.195 |
| **Anterior circulation aneurysm** | 207 | 30.133 | 0.889 | 28.391 | 31.876 |  |  |
| **Posterior circulation aneurysm** | 9 | 19.962 | 4.252 | 11.628 | 28.296 |  |  |
| **Aneurysm size** |  |  |  |  |  | 8.722 | 0.013 * |
| **0~0.5cm** | 82 | 30.569 | 1.352 | 27.919 | 33.219 |  |  |
| **0.5~1.5cm** | 109 | 30.656 | 1.177 | 28.349 | 32.962 |  |  |
| **1.5cm~2.5cm** | 24 | 16.179 | 2.642 | 11.000 | 21.357 |  |  |
| **≥2.5cm** | 1 | . | . | . | . |  |  |
| **Surgery** |  |  |  |  |  | 4.165 | 0.041 * |
| **Embolism** | 77 | 31.253 | 1.101 | 29.096 | 33.411 |  |  |
| **Clipping** | 139 | 28.477 | 1.207 | 26.111 | 30.844 |  |  |
| **Hydrocephalus** |  |  |  |  |  | 53.610 | ＜0.001 ** |
| **No** | 161 | 33.394 | 0.743 | 31.937 | 34.851 |  |  |
| **Yes** | 55 | 17.254 | 1.842 | 13.643 | 20.864 |  |  |
| **DCI** |  |  |  |  |  | 25.276 | ＜0.001 ** |
| **No** | 124 | 33.789 | 0.804 | 32.214 | 35.365 |  |  |
| **Yes** | 92 | 25.019 | 1.585 | 21.912 | 28.125 |  |  |
| *P < 0.05; **P < 0.01 |  |  |  |  |  |  |  |

## 2 Supplementary Figures


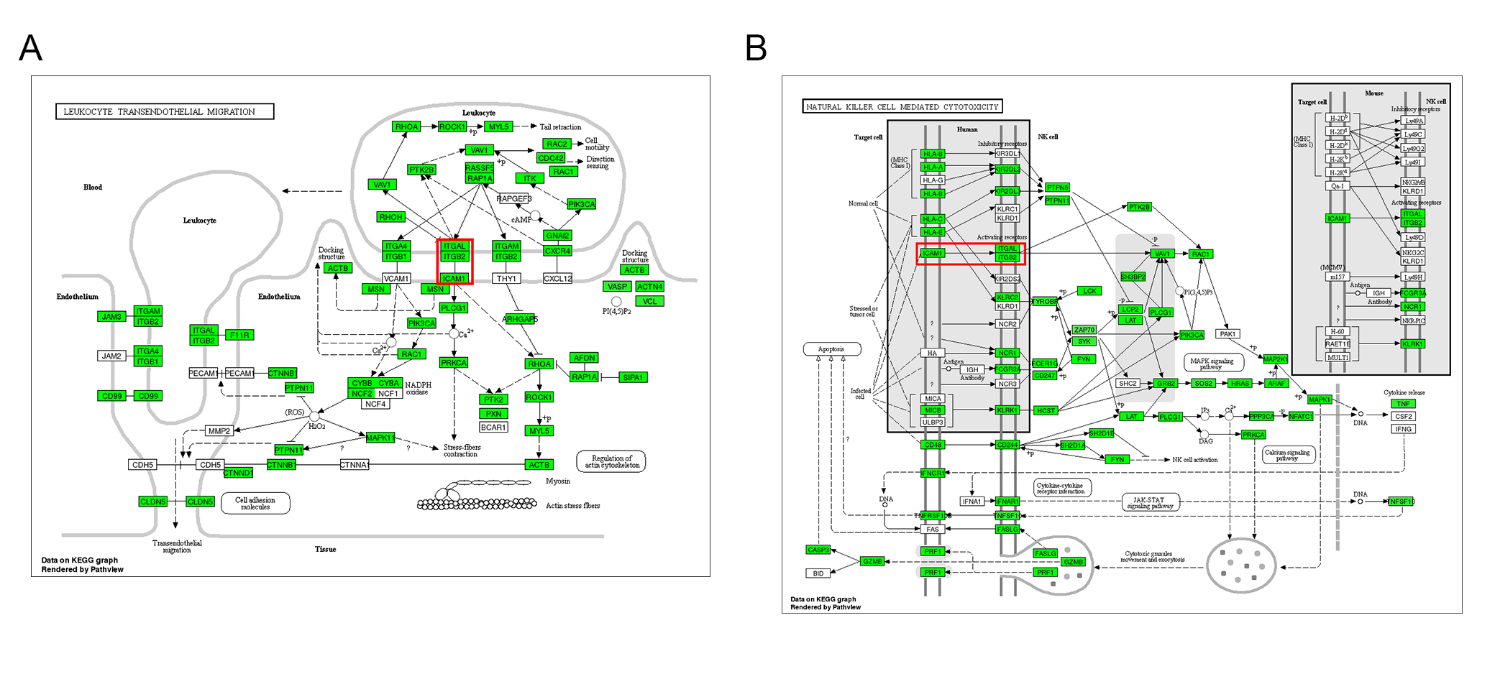


**Supplementary Figure 1.** Pathways associated with the formation of IA. (A) Leukocyte transendothelial migration pathway. (B) Natural killer cell mediated cytotoxicity pathway. Green nodes represent gene enrichment of the pathway, while white nodes have no significance. The red square represents adhesion between intercellular cell adhesion molecule‑1 and the complex of integrin subunit αL/integrin subunit β2.
